# FYN Tyrosine Kinase Gene Polymorphisms in Alcohol-Dependent Korean Patients

**DOI:** 10.31083/AP38752

**Published:** 2025-02-28

**Authors:** Sung Young Huh, Sung-Gon Kim, Ji-Hoon Kim, Hyeon-Kyeong Kim, Yeon-Sue Kim

**Affiliations:** ^1^Department of Psychiatry, Pusan National University Yangsan Hospital, 50612 Yangsan, Republic of Korea; ^2^Department of Psychiatry, Pusan National University School of Medicine, 46639 Yangsan, Republic of Korea; ^3^Medical Research Institute, Pusan National University, 50612 Yangsan, Republic of Korea

**Keywords:** *FYN*, gender differences, gene polymorphism, alcohol dependence

## Abstract

**Background:**

Alcohol use disorder (AUD) is a common disease with a high economic cost. The glutamate cell signaling pathway associated with alcohol has been reported to be one of the main pathologies of AUD. Previous studies have suggested that *FYN*, which is known to control NMDA glutamate receptor function through phosphorylation, might be associated with AUD.

**Method:**

The present study included 354 subjects in the alcohol-dependent group and 139 subjects in the control group. The alcohol-dependent group was recruited from five university hospitals and a psychiatric hospital, and the control group was recruited from people who visited the university hospital for routine medical checkups in Korea. *FYN *gene single nucleotide polymorphism (SNPs) were selected based on SNP databases and previous studies of the *FYN* gene. Ten SNPs were genotyped using polymerase chain reaction-restriction fragment length polymorphism techniques.

**Results:**

GG genotypes and G allele frequencies of rs1058134 in male AUD patients were significantly lower than in controls (*p* = 0.003). AA genotypes and A allele frequencies of rs12191154 in female AUD patients were significantly lower than in controls (*p* < 0.001, *p* = 0.003). In female AUD patients, AA genotypes and A allele frequencies of rs9387025 were significantly higher than in controls (*p* = 0.003).

**Conclusion:**

These findings suggest that the *FYN* gene may be a candidate gene for AUD. This may help for the planning of further studies to determine the function of each SNP and the exact relationship between the *FYN* gene and AUD.

## Main Points

• There were significant differences in *FYN* genotype and allele frequencies between the alcohol-dependent group and the normal control group.

• There were also significant differences in the genotype and allele frequencies of different SNPs between males and females.

• The *FYN* gene might be related to the occurrence of alcohol use disorder.

• This study suggests that consideration of sex differences is necessary when studying *FYN* gene polymorphisms.

## 1. Introduction

Alcohol use disorder is caused by a variety of neurobiological, social, psychological, and environmental factors [1, 2]. Of these, glutamate and related neurotransmitters are considered to be major causes of alcohol use disorders. They are not only related to changes in the brain after alcohol consumption, but also related to brain damage caused by continuous alcohol use [3, 4]. Terranova *et al*. [5] suggested an association between polymorphism in the GAD67 (glutamate decarboxylase 67) gene and alcohol dependence in Italian men. Xia *et al*. [6] have found that polymorphism in the metabolite glutamate receptor GRM3 (glutamate receptor 3) gene is associated with the occurrence of alcohol dependence. Kranzler *et al*. [7] have also demonstrated that polymorphism in GRIK1 (GluR5 Kainate Receptor Subunit gene), a kainate receptor in the glutamate ion receptor family, contributes to the risk of alcohol dependence.

The N-methyl-D-aspartate (NMDA) receptor is an ionic glutamate receptor known to play an essential role in synaptic plasticity and learning memory [8, 9]. Previous studies have shown that the NMDA glutamate receptor is a major target when alcohol acts on the brain and that it is associated with alcohol tolerance, withdrawal, and sensitivity [10, 11]. The function of the NMDA glutamate receptor is balanced by the activities of serine/threonine kinase and phosphatase, and tyrosine kinase and phosphatase [12, 13]. *FYN* tyrosine kinase, a member of the Src family of kinases, is known to control NMDA glutamate receptor function through phosphorylation [14]. In particular, phosphorylation of NR2B (NMDA receptor 2b), a subunit of the NMDA receptor, upregulates the function of the ion channel [15] and reduces the inhibitory effect of ethanol, leading to differences in sensitivity to ethanol. According to Miyakawa *et al*. [16], *FYN* knockout mice show increased sensitivity to ethanol and decreased acute resistance response to ethanol compared with control mice. In addition, through a study using *FYN* kinase null mutant mice, Boehm *et al*. [17] have suggested that *FYN* kinase can regulate acute tolerance to ethanol and mediate the anti-anxiety effect and negative reinforcement of ethanol.

A study of *FYN* gene polymorphism in humans by Schumann *et al*. [18] has reported that T137346C (C risk allele, rs45490695), a single nucleotide polymorphism (SNP) in *FYN*, is associated with alcohol dependence in Spanish men. Pastor *et al*. [19] have also found that the same SNP can increase the risk of developing alcohol dependence compared with alcohol abuse in Spanish men. However, a study by Ishiguro *et al*. [20] on Japanese did not find a significant association between this SNP and alcohol dependence. These studies, however, only compared an alcohol-dependent group with a normal control group without comparing subjects by sex.

Previous studies have suggested that the mechanism for the occurrence of alcohol use disorder may differ depending on sex and that there might be genetic differences according to sex for the occurrence of alcohol use disorder [21, 22, 23]. In other words, it is widely known that alcohol metabolism differs between men and women and that these difference are affected by genetic factors [22, 24]. Previous studies have also shown that the risk for alcohol use disorder may differ between men and women [25, 26]. Prescott *et al*. [27] have also suggested that genetic factors play an important role in the development of alcohol use disorder and that genetic vulnerability is not the same in men and women. Taken together, it seems that the mechanism of occurrence of alcohol use disorder and the risk for alcohol use disorder may differ according to sex.

Based on results of previous studies, glutamate is an important neurotransmitter for the occurrence and maintenance of alcohol use disorder. The NMDA glutamate receptor is regulated by *FYN* tyrosine kinase. *FYN* kinase is sensitive to ethanol and associated with the development of an acute tolerance reaction to ethanol, which is thought to be directly or indirectly related to the development of alcohol use disorder. This suggests that alcohol use disorder might be associated with *FYN* tyrosine kinase gene polymorphism. However, there have been only three studies on genetic polymorphism of the *FYN* tyrosine kinase in humans (one in Caucasians, one in Spaniards, and one in Japanese) regardless of sex. Such studies on Koreans have not been reported yet. There are no such studies considering sex differences. Thus, the aim of this study was to investigate differences in the frequencies of *FYN* gene SNPs between alcohol-dependent patients and normal controls according to sex.

## 2. Methods

### 2.1 Subjects

The patient group (alcohol dependence (AD) group) were diagnosed with alcohol dependence by psychiatrists using the Diagnostic and Statistical Manual of Mental Disorders, Fourth Edition (DSM-IV). Those who had other major psychiatric disorders (e.g., schizophrenia, affective disorder) or another substance use disorder (except nicotine use disorder or caffeine use disorder) were excluded. The alcohol dependence group comprised 354 subjects (279 males and 75 females). The AD group was recruited from seven university hospitals and a psychiatric hospital in Pusan and KyungNam in Korea from 1997 to 2017.

The normal control group were individuals who attended the university hospital for a routine health check-up in Pusan from 1995 to 2000. We defined the normal control group according to the method suggested by Town *et al*. [28]. We defined the normal control group as subjects who were exposed to sufficient alcohol but did not develop pathological drinking habits. We included subjects who were aged 50 years or older and consumed less than five standard drinks (SD) per month, which was considered sufficient exposure to alcohol. We excluded those who had never drunk alcohol. The normal control group comprised 139 subjects (males 80 and females 59). We collected the venous blood of subjects who fasted for more than 12 hours.

### 2.2 Separation and Purification of DNA

An EZNA Blood DNA kit (Omega Biotech Int., Norcross, GA, USA, SKU#D2492-03) was used to extract genomic DNA from blood samples. One milliliter of cell lysis buffer (320 mM sucrose, 1% (v/v) Triton X-100, 5 mM MgCl_2_, 10 mM Tris-HCl; pH 7.6) was added to 500 µL whole blood. The mixture was vortexed and centrifuged at 10,000 rpm for 1 minute. The pellet was washed with 250 µL phosphate-buffered saline (PBS) and vortex-mixed with 25 µL proteinase (20 mg/mL) and 250 µL buffer BL. The mixture was incubated at 70 °C for 10 minutes and vortex-mixed several times. Isopropanol (260 µL) was then added to the lysate and mixed. The lysate was transferred to a HiBind DNA Spin column and centrifuged at 10,000 rpm for 1 minute. The collected fluid was discarded and the column was washed twice with 750 µL 80% ethanol, followed by centrifugation at 13,000 rpm for 3 minutes and air dried. Finally, genomic DNA was eluted with 200 µL elution buffer at 70 °C from the column. The quantity and purity of extracted genomic DNA were estimated by resolving on a 1% agarose gel using EtBr staining. DNA samples were then stored at –20 °C.

### 2.3 FYN Gene SNPs Analysis

**Fig. 1.**
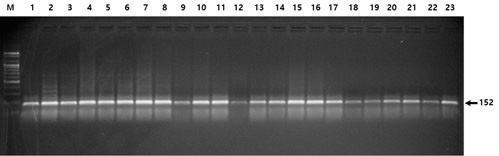
**Electrophoresis of the PCR products digested with the restriction enzyme *DdeI *(rs559963242)**. All lanes indicate homozygosity for the more common allele (TT); heterozygosity (TG) and homozygosity for the less common allele (GG) are not shown.

**Fig. 2.**
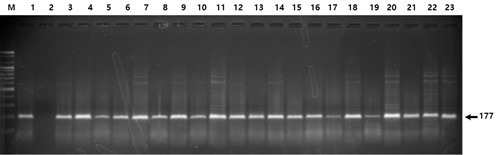
**Electrophoresis of the PCR products digested with the restriction enzyme *BsmAI *(rs376330544)**. All lanes indicate homozygosity for the more common allele (CC); heterozygosity (CT) and homozygosity for the less common allele (TT) are not shown.

**Fig. 3.**
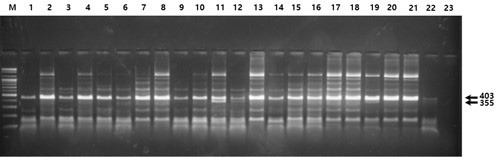
**Electrophoresis of the PCR products digested with the restriction enzyme *Tsp45I *(rs12191154)**. All lanes except lanes 3, 10, 11, 12, 19, and 22 indicate homozygosity for the more common allele (AA); lanes 3, 10, 11, 12, 19, and 22 indicate heterozygosity (AG); homozygosity for the less common allele (GG) is not shown.

*FYN* SNPs were identified from searching the NCBI SNP database and previous studies about the *FYN* gene which showed significant SNP frequencies in Asians [18, 19, 20]. Ten SNPs (rs559963242, rs376330544, rs12191154, rs9387025, rs3730353, rs752601385, rs1058134, rs11967460, rs9481198, and rs62413757) were selected. These SNPs were able to be typed using the polymerase chain reaction-restriction fragment length polymorphism (PCR-RFLP) technique. Table 1 shows the locations of these SNPs within the *FYN* gene. All were located in introns. The PCR reactions consisted of 1.5 mM MgCl_2_, 200 µM dNTP (dATP, dCTP, dGTP, and dTTP), and 0.4 µM of reverse and forward primers for each SNP (Table 2). The amplification products had the following sizes: rs559963242 and rs376330544, 203 bp; rs12191154 and rs9387025, 403 bp; rs3730353 and rs752601385, 463 bp; rs1058134, 280 bp; rs11967460, 306 bp; rs9481198, 300 bp; and rs62413757, 232 bp. The amplified *FYN* gene products were then subjected to the RFLP (restriction fragment length polymorphism) technique. The amount of missing data was small and did not affect the main results of this study. The rs1058134 (280 bp) amplified product was incubated with 2.5 units of *PstI* endonuclease at 37 °C for 3 hours. The band patterns were assessed after 2.5% agarose gel electrophoresis at 200 V for 2 hours. rs1058134 genotyping products were interpreted as follows: 117 bp and 107 bp, GG; 224 bp, 117 bp, and 107 bp, GT; and 224 bp, TT. We analyzed other SNPs using the same technique. Figs. 1,2,3 show examples of the electrophoresis patterns for three SNPs.

**Table 1.** **Alleles and positions of the FYN gene SNPs analyzed in this study**.

SNP ID	Chromosome position (GRCh38)	Region	Alleles
rs559963242	111661517	3′ Prime UTR	T>G
rs376330544	111661540	3′ Prime UTR	C>T
rs12191154	111663112	Intron	A>G
rs9387025	111663197	Intron	G>A
rs3730353	111674463	Intron	A>G
rs752601385	111674583	Exon	C>G
rs1058134	111700152	Exon	G>T
rs11967460	111794545	Intron	G>A
rs9481198	111871592	Intron	T>C
rs62413757	111872133	Intron	G>A

GRCh38, Genome Reference Consortium Human Genome build 38; SNP, single nucleotide polymorphism; UTR, untranslated region.

**Table 2.** **Primer sequences for initial PCR amplification**.

SNP ID	Primer	Primer Sequence	PCR Tm (°C)	PCR product size (bp)	Restriction enzyme
rs559963242	Forward	AGTTGAATCAGGTGAAGACA	54	203	*DdeI*
	Reverse	AATCCGAACCTCCTCTGT			
rs376330544	Forward	AGTTGAATCAGGTGAAGACA	54	203	*BsmAI*
	Reverse	AATCCGAACCTCCTCTGT			
rs12191154	Forward	AACTACTGCCAGCCACTA	54	403	*Tsp45I*
	Reverse	GTAACAACACAACCAAGAGG			
rs9387025	Forward	AACTACTGCCAGCCACTA	54	403	*BsrI*
	Reverse	GTAACAACACAACCAAGAGG			
rs3730353	Forward	TGGCTGCTTGTCTACATAC	54	463	*DpnI*
	Reverse	TTCCTGCTTCACCTCCTT			
rs752601385	Forward	TGGCTGCTTGTCTACATAC	54	463	*MspI*
	Reverse	TTCCTGCTTCACCTCCTT			
rs1058134	Forward	CATCTTTGGTGTTTGGGAT	52	280	*PstI*
	Reverse	ATGTGTTCTGCTCTTCTCT			
rs11967460	Forward	ACACAGAGGAATCACAAGG	54	306	*PstI*
	Reverse	TTTACTTACGGGCACTGAG			
rs9481198	Forward	GGGTGGTGAGAAAGGAAG	54	300	*RsaI*
	Reverse	GGTAGAGTAGATTGGTGTGG			
rs62413757	Forward	GTCACACTCACACTCACA	53	232	*MluCI*
	Reverse	GATTTCCTCTCTACTTCCCTA			

PCR, polymerase chain reaction; SNP, Single Nucleotide Polymorphism; Tm, temperature; bp, base pair.

### 2.4 Statistical Analysis

Descriptive statistics were used to summarize baseline and clinical characteristics of patients. The normality test was performed on continuous variables and the Chi-squared test was used for categorical variables. Genotype distributions were tested for Hardy-Weinberg equilibrium (HWE) using the Chi-squared test.

SNP genotype and allele frequency (rs559963242, rs376330544, rs12191154, rs9387025, rs3730353, rs752601385, rs1058134, rs11967460, rs9481198, and rs62413757) distributions were compared between the patient group and the normal group using Fisher’s exact test or the Chi-squared test. Logistic regression analysis was performed on significant variables. Statistical significance was set at *p* < 0.05. All statistical analyses were carried out using IBM SPSS Statistics for Windows, version 26.0 (Armonk, NY, USA: IBM Corp).

## 3. Results

### 3.1 Clinical Characteristics and Demographic Data of the AD Patient Group

The patient group had a mean age of 47.24 ± 9.43 years. The average age of first drinking was 20.78 ± 6.30 years. The age of onset of drinking in female AD patients was significantly higher than in male AD patients (*p* < 0.001). Alcohol-related problems (ARP) started at the age of 34.00 ± 10.55 years. Female AD patients had ARP significantly (*p* = 0.030) later than male AD patients. The average number of hospitalizations for ARP per subject was 4.90 ± 6.65. Female AD patients had significantly less admission numbers (*p* = 0.001) than male AD patients. About 55% of AD patients had severe alcohol withdrawal histories, and male AD patients had a significantly lower incidence of severe alcohol withdrawal histories than female AD patients (51.3% vs 70.7%, *p* = 0.003). There were no significant differences in age at first hospitalization, family history of ARP, drinking days/month, or education level between male and female patients (see Table 3).

**Table 3.** **Clinical characteristics and demographic data of alcohol-dependent patients**.

	Total (N = 354)	Male (N = 279)	Female (N = 75)	*t *or χ^2^	*p*-value
Age (years)	47.24 ± 9.43	47.97 ± 9.29	44.53 ± 9.53	2.83	**0.005**
Education (years)	10.34 ± 4.18	10.51 ± 4.06	9.70 ± 4.54	1.50	0.135
First drinking age (years)	20.78 ± 6.30	19.53 ± 4.38	25.40 ± 9.48	–5.21	< **0.001**
Onset age of ARP (years)	34.00 ± 10.55	33.37 ± 10.65	36.34 ± 9.90	–2.18	**0.030**
First hospitalization age (years)	41.85 ± 9.58	41.80 ± 9.69	42.04 ± 9.23	–0.19	0.852
Number of hospitalization due to ARP	4.90 ± 6.65	5.35 ± 7.30	3.24 ± 2.74	3.92	< **0.001**
Drinks/drinking day (SD)^†^	12.75 ± 7.49	13.08 ± 7.50	11.51 ± 7.36	1.67	0.108
Drinking days/month^‡^	16.70 ± 9.20	17.50 ± 9.55	15.88 ± 8.85	1.33	0.186
Family history of ARP	225 (63.6%)	178 (63.8%)	47 (62.7%)	0.95	0.893
History of severe alcohol withdrawal	196 (55.4%)	143 (51.3%)	53 (70.7%)	2.29	**0.003**

ARP, alcohol related problems (e.g., drunk driving, legal problems due to drinking, family troubles due to drinking); SD, standard drink; †, Average daily standard drinking amounts for 1 year prior to current admission; ‡, Average drinking days/month of 1 year prior to current admission; bold: *p* < 0.05.

### 3.2 FYN Gene SNP Genotypes in the Patient Group and the Normal Control Group

No allele or genotype frequencies violated HWE. Table 4 shows the distribution of *FYN* gene SNP genotypes in the AD patient group and the normal control group. The major allele GG genotype and G allele frequencies of rs1058134 in the AD patient group were significantly lower than those in the normal control group (genotype frequency: *p* < 0.001; allele frequency: *p* = 0.003). Genotype and allele frequencies of other SNPs did not show significant differences between patients and normal controls. Table 5 shows the effect of genotype on AD after adjusting for sex using multivariate logistic regression analysis. The GG genotype of rs1058134 showed a significant odds ratio of 0.50 (95% CI: 0.32–0.76) compared with the GT+GG genotype.

**Table 4.** **Distribution of the FYN gene SNP rs559963242, rs376330544, rs62413757, rs3730353, rs752601385, rs12191154, rs9387025, rs1058134, rs11967460, and rs9481198 genotypes in alcohol-dependent patients (AD) and normal controls (NC)**.

SNP	Sample	n	Distribution of Genotypes (%)			*p*-value (χ^2^)	Genotype Frequency (%)		*p*-value (χ^2^)	Allele Frequency (%)		*p*-value (χ^2^)
rs559963242			TT				TT			*f(T)*		
	AD	346	346	0	0	-	346	0	-	692	0	
			(100.0)	(0.0)	(0.0)		(100.0)	(0.0)		(100.0)	(0.0)	
	NC	137	137	0	0		137	0		274	0	
			(100.0)	(0.0)	(0.0)		(100.0)	(0.0)		(100.0)	(0.0)	
rs376330544			CC				CC			*f(C)*		
	AD	346	346	0	0	-	346	0	-	692	0	
			(100.0)	(0.0)	(0.0)		(100.0)	(0.0)		(100.0)	(0.0)	
	NC	137	137	0	0		137	0		274	0	
			(100.0)	(0.0)	(0.0)		(100.0)	(0.0)		(100.0)	(0.0)	
rs3730353			AA				AA			*f(A)*		
	AD	337	337	0	0	-	337	0	-	674	0	
			(100.0)	(0.0)	(0.0)		(100.0)	(0.0)		(100.0)	(0.0)	
	NC	137	137	0	0		137	0		274	0	
			(100.0)	(0.0)	(0.0)		(100.0)	(0.0)		(100.0)	(0.0)	
rs752601385			CC				CC			*f(C)*		
	AD	337	337	0	0	-	337	0	-	674	0	-
			(100.0)	(0.0)	(0.0)		(100.0)	(0.0)		(100.0)	(0.0)	
	NC	137	137	0	0		137	0		274	0	
			(100.0)	(0.0)	(0.0)		(100.0)	(0.0)		(100.0)	(0.0)	
rs62413757			GG				GG			*f(G)*		
	AD	344	344	0	0	-	344	0	-	688	0	-
			(100.0)	(0.0)	(0.0)		(100.0)	(0.0)		(100.0)	(0.0)	
	NC	137	137	0	0		137	0		274	0	
			(100.0)	(0.0)	(0.0)		(100.0)	(0.0)		(100.0)	(0.0)	
rs12191154			AA	AG	GG		AA	AG+GG		*f(A)*	*f(G)*	
	AD	327	145	176	6	0.173 (3.32)	145	182	0.078 (3.10)	466	188	0.117 (2.46)
			(44.3)	(53.8)	(1.9)		(44.3)	(55.7)		(71.3)	(28.7)	
	NC	137	73	63	1		73	64		209	65	
			(53.3)	(46.0)	(0.7)		(53.3)	(46.7)		(76.3)	(23.7)	
rs9387025			AA	GA	*GG*		AA	GA+GG		*f(A)*	*f(G)*	
	AD	336	219	111	6	0.273 (2.60)	219	117	0.125 (2.36)	549	123	0.128 (2.31)
			(65.2)	(33.0)	(1.8)		(65.2)	(34.8)		(81.7)	(18.3)	
	NC	137	79	54	4		79	58		212	62	
			(57.7)	(39.4)	(2.9)		(57.7)	(42.3)		(77.4)	(22.6)	
rs1058134			GG	GT	TT		GG	GT+TT		*f(G)*	*f(T)*	
	AD	347	154	193	0	< **0.001 (13.29)**	154	193	< **0.001 (13.29)**	501	193	**0.003 (8.81)**
			(44.4)	(55.6)	(0.0)		(44.4)	(55.6)		(72.2)	(27.8)	
	NC	137	86	51	0		86	51		223	51	
			(62.8)	(37.2)	(0.0)		(62.8)	(37.2)		(81.4)	(18.6)	
rs11967460			GG	GA	AA		GG	GA+AA		*f(G)*	*f(A)*	
	AD	348	238	96	14	0.050 (6.01)	238	110	0.962 (0.002)	572	124	0.430 (0.62)
			(68.4)	(27.6)	(4.0)		(68.4)	(31.6)		(82.2)	(17.8)	
	NC	137	94	43	0		94	43		231	43	
			(68.6)	(31.4)	(0.0)		(68.6)	(31.4)		(84.3)	(15.7)	
rs9481198			TT	TC	CC		TT	TC+CC		*f(T)*	*f(C)*	
	AD	350	107	187	56	0.259 (2.71)	107	243	0.113 (2.51)	401	299	0.304 (1.06)
			(30.6)	(53.4)	(16.0)		(30.6)	(69.4)		(57.3)	(42.7)	
	NC	137	32	83	22		32	105		147	127	
			(23.4)	(60.6)	(16.0)		(23.4)	(76.6)		(53.6)	(46.4)	

bold: *p* < 0.05.

**Table 5.** **Multivariate logistic regression analysis of the effect of genotype on alcohol dependence**.

Variable		Odds Ratio	(95% CI)	*p*-value	Wald χ^2^	Estimate	SE
Sex	Female	1.000					
	Male	2.38	(1.53–3.70)	<0.001	14.834	0.867	0.225
rs11967460	GA+AA	1.00					
	GG	0.95	(0.60–1.51)	0.817	0.054	–0.055	0.237
rs9481198	TC+CC	1.00					
	TT	1.45	(0.88–2.37)	0.142	2.160	0.370	0.252
rs1058134	GT+TT	1.00					
	GG	0.50	(0.32–0.76)	0.001	10.345	–0.700	0.218
rs12191154	AG+GG	1.00					
	AA	0.74	(0.49–1.13)	0.164	1.941	–0.299	0.214
rs9387025	GA+GG	1.00					
	AA	1.20	(0.78–1.85)	0.407	0.686	0.183	0.221

SE, stand error.

### 3.3 Distributions of FYN Gene SNP Genotypes in the AD Patient Group and the Normal Control Group by Sex

Table 6 shows the distribution of SNP genotypes of the *FYN* gene in male and female subjects of patients and normal controls. The major AA genotype and A allele frequencies and of rs12191154 in female patients were significantly lower than in normal female controls (allele frequency: *p* = 0.003, genotype frequency: *p* < 0.001). However, allele frequencies and genotypes of rs12191154 in males did not show differences between patients and normal controls. The major allele AA genotype and A allele frequencies of rs9387025 in female AD patients were also significantly higher than those in normal female controls (genotype frequency: *p* = 0.001; allele frequency: *p* = 0.003). However, genotype and allele frequencies of rs9387025 in males did not differ between AD patients and normal controls. In contrast, the major allele GG genotype and G allele frequencies of rs1058134 in male patients were lower compared with normal controls (allele frequency: *p* = 0.035, genotype frequency: *p* = 0.008). However, allele frequencies and genotypes of rs1058134 did not show significant differences between female patients and normal female controls.

**Table 6.** **Distribution of the FYN gene SNP rs12191154, rs9387025, rs1058134, rs11967460, and rs9481198 genotypes between males and females of the alcohol-dependent (AD) group and normal control (NC) group**.

SNP	Sample		n	Distribution of Genotypes (%)			*p*-value (χ^2^)	Genotype Frequency (%)		*p*-value (χ^2^)	Allele Frequency (%)		*p*-value (χ^2^)
rs12191154				AA	AG	GG		AA	AG+GG		*f(A)*	*f(G)*	
	M	AD	254	116	133	5	0.299^§^ (2.70)	116	138	0.357 (0.85)	365	143	0.632 (0.23)
				(45.7)	(52.3)	(2.0)		(45.7)	(54.3)		(71.9)	(28.1)	
		NC	78	31	47	0		31	47		109	47	
				(39.7)	(60.3)	(0.0)		(39.7)	(60.3)		(69.9)	(30.1)	
	F	AD	73	29	43	1	< **0.001^§^ (13.76)**	29	44	< **0.001 (12.99)**	101	45	**0.003 (8.71)**
				(39.7)	(58.9)	(1.4)		(39.7)	(60.3)		(69.2)	(30.8)	
		NC	59	42	16	1		42	17		100	18	
				(71.2)	(27.1)	(1.7)		(71.2)	(28.8)		(84.7)	(15.3)	
rs9387025				AA	GA	*GG*		AA	GA+GG		*f(A)*	*f(G)*	
	M	AD	263	166	94	3	0.682^§^ (2.53)	166	97	0.434 (0.61)	426	100	0.397 (0.72)
				(63.1)	(35.7)	(1.2)		(63.1)	(36.9)		(81.0)	(91.0)	
		NC	78	53	25	0		53	25		131	25	
				(67.9)	(32.1)	(0.0)		(67.9)	(32.1)		(84.0)	(16.0)	
	F	AD	62	53	17	3	**0.003^§^ (11.15)**	53	20	**0.001 (11.06)**	123	23	**0.003 (9.05)**
				(72.6)	(23.3)	(4.1)		(72.6)	(27.4)		(84.2)	(15.8)	
		NC	59	26	29	4		26	33		81	37	
				(44.1)	(49.2)	(6.8)		(44.1)	(55.9)		(68.6)	(31.4)	
rs1058134				GG	GT	TT		GG	GT+TT		*f(G)*	*f(T)*	
	M	AD	272	114	158	0	**0.008 (7.11)**	114	158	**0.008 (7.11)**	386	158	**0.035 (4.46)**
				(41.9)	(58.1)	(0.0)		(41.9)	(58.1)		(71.0)	(29.0)	
		NC	78	46	32	0		46	32		124	32	
				(59.0)	(41.0)	(0.0)		(59.0)	(41.0)		(79.5)	(20.5)	
	F	AD	75	40	35	0	0.090 (2.87)	40	35	0.090 (2.87)	115	35	0.143 (2.15)
				(53.3)	(46.7)	(0.0)		(53.3)	(46.7)		(76.7)	(23.3)	
		NC	59	40	19	0		40	19		99	19	
				(67.8)	(32.2)	(0.0)		(67.8)	(32.2)		(83.9)	(16.1)	
rs11967460				GG	GA	AA		GG	GA+AA		*f(G)*	*f(A)*	
	M	AD	274	185	79	10	0.230 (2.94)	185	89	0.616 (0.25)	449	99	0.333 (0.94)
				(67.5)	(28.8)	(36)		(67.5)	(32.5)		(81.9)	(18.1)	
		NC	78	55	23	0		55	23		133	23	
				(70.5)	(29.5)	(0.0)		(70.5)	(29.5)		(85.3)	(14.7)	
	F	AD	74	53	17	4	0.108 (4.74)	53	32	0.493 (0.47)	123	25	0.990 (0.00)
				(71.6)	(23.0)	(5.4)		(71.6)	(28.4)		(83.1)	(16.9)	
		NC	59	39	20	0		39	20		98	20	
				(66.1)	(33.9)	(0.0)		(66.1)	(33.9)		(83.1)	(16.9)	
rs9481198				TT	TC	CC		TT	TC+CC		*f(T)*	*f(C)*	
	M	AD	277	83	149	45	0.268 (2.63)	83	194	0.234 (1.42)	315	239	0.700 (0.15)
				(30.0)	(53.8)	(16.2)		(30.0)	(70.0)		(56.9)	(43.1)	
		NC	78	18	50	10		18	60		86	70	
				(23.1)	(64.1)	(12.8)		(23.1)	(76.9)		(55.1)	(44.9)	
	F	AD	73	24	38	11	0.458 (1.56)	24	49	0.248 (1.33)	86	60	0.241 (1.37)
				(32.9)	(52.0)	(15.1)		(32.9)	(67.1)		(58.9)	(41.1)	
		NC	59	14	33	12		14	45		61	57	
				(23.7)	(55.9)	(20.3)		(23.7)	(76.3)		(51.7)	(48.3)	

^§^: Fisher’s exact test; M, male; F, female; bold: *p* < 0.05.

## 4. Discussion

In the present study, allele frequencies and genotypes of *FYN* gene SNPs were compared between AD patients and normal controls. Allele frequencies and genotypes of rs1058134 showed significant differences between the AD group and the normal control group. In the second analysis, allele frequencies and genotypes of rs1058134 did not show significant differences between patients and normal controls in males, while allele frequencies and genotypes of rs12191154 and rs9387025 did not show significant differences between female patients and normal female controls. The major allele genotypes of rs1058134 showed significantly lower odds ratios for alcohol dependence than minor allele genotypes in a logistic regression analysis adjusted for sex.

Few studies have previously reported associations between polymorphisms in the *FYN* gene and alcohol dependence. In a study of Caucasian subjects by Schumann *et al*. [18], those with major allele genotypes of rs45490695 showed a high number of withdrawal symptoms, high amount of alcohol intake, and high maximum number of drinks than those with minor genotypes of alcohol dependence. showed a high number of withdrawal symptoms, high amount of alcohol intake, and high maximum number of drinks than those with minor genotypes of alcohol dependence. In a study of Spanish subjects by Pastor *et al*. [19], G allele carriers of rs45490695 were more common in those with alcohol dependence than in those with alcohol abuse. However, in a study of Japanese subjects by Ishiguro *et al*. [20], alcohol dependence showed no significant association with *FYN* gene SNPs such as rs45490695, rs3730353, or rs6916861. Previous studies have reported that there might be differences in sensitivity to alcohol and alcohol metabolism depending on race [29, 30, 31]. These factors could eventually affect the occurrence and risk of alcohol use disorders.

In two previous studies, there were racial differences between the Caucasian and Spanish subjects and Korean subjects. In a previous study of Japanese subjects, rs3730353, which failed to reveal a significant association, showed the same genotype in both the alcohol dependence group and the control group in this study. Although SNP rs45490695 showed significant association in previous studies, it could not be included in present study because there was no appropriate enzyme restriction site for genotyping. This was a limitation of the present study.

In addition, in the study of Ishiguro *et al*. [20], the control group was 29–75 years old (average 48.3 years). In the study of Schumann *et al*. [18], the control group was 44.5 years old on average. In both studies, there was no standard for alcohol consumption. However, subjects who were alcohol dependent might have been included in the present study, which could have affected the results. In a study by Schinka *et al*. [32], the frequency of A118G genotypes in OPRM1 (opioid acceptor Mu1) did not show a significant difference compared with those in the normal control group. However, results can differ depending on the control group, e.g., a super control group with an average monthly drinking rate of lower than 1 SD and less than 1 cigarette per week [32]. This supports the need for sufficient consideration of alcohol consumption standards and age when creating the control group.

In this study, referring to study by Town *et al*. [28], we defined the control group as those who did not develop alcohol dependence at age 50 years or older, who had sufficient exposure to alcohol, to exclude subjects with the potential to develop alcohol dependence. Compared with previous studies, this could be a strength of the present study.

SNPs such as rs1058134, rs12191152, and rs9387025 showed a significant difference in this study. However, they did not show associations with alcohol dependence in prior studies, although their associations with Alzheimer’s disease have been suggested by Zahratka *et al*. [33] and Anbarasu and Kundu [34]. Many previous studies have demonstrated the relationship between polymorphism in the *FYN* gene and phosphorylation of Tau protein, one important etiology for Alzheimer’s disease [35, 36, 37]. Although there are few studies on alcohol use disorder and phosphorylation of Tau protein, Saito *et al*. [38] have demonstrated that ethanol can induce phosphorylation of Tau protein which might be related to ethanol-induced neurodegeneration in the brain.

It is known that drinking patterns, drinking experiences, severity of alcohol withdrawal, and frequency of alcohol problems differ according to sex. Factors affecting the occurrence of alcohol use disorders are also different between men and women [39]. In female mice exposed to alcohol for a long time, the expression of NR1, an NMDA acceptor subreceptor, was increased in the cerebral envelope and hippocampus, whereas in male mice, the expression of NR1 was increased only in the hippocampus. In addition, in the case of NR2A, its expression was increased only in the hippocampus of male mice exposed to alcohol for a long time. In the case of NR2B, its expression was increased in the cerebral cortex of both males and females [40]. *FYN* kinase adjusts the NMDA-type glutamate receptor function through phosphorylation [14]. It can increase the function of ion passage through phosphorylation of NR2B, the subunit of the NMDA receptor [15], induce the integrity effect of ethanol, and cause a difference in alcohol sensitivity. Previous studies have not been conducted in humans, so it is difficult to consider them as sufficient evidence for the sex differences found in this study. However, this could be seen as evidence for suggesting that there may be sex differences when the *FYN* kinase regulates NMDA receptor function, and it could be helpful for future research plans on how the mechanism of alcohol use disorder in humans differs between males and females.

This study has some limitations. First, the number of women in the alcohol-dependent group was relatively small compared with the number of men in the same group. Second, in the SNP selection process, specific SNPs were selected and implemented based on previous studies rather than the whole genome sequencing method. In particular, rs45490695, a SNP used in previous studies, was not included in the analysis because there was no appropriate restriction site. Therefore, additional studies should be planned to verify results of this study with more SNPs, including outpatients and female patients with alcohol use disorder.

## 5. Conclusion

There were significant differences in genotype frequency and allele frequency of *FYN* gene SNPs between the alcohol dependence group and the normal control group. There were also significant differences in genotype frequency and allele frequency of different SNPs between males and females. The results of this study suggest that the *FYN* gene might be related to the occurrence of alcohol use disorder. Therefore, additional research is needed to confirm the relationship between the *FYN* gene and alcohol use disorder in each sex through an accurate study of the function of the meaningful SNPs described in this study.

## Availability of Data and Materials

The data sets generated and/or analyzed during the study are available at https://www.ncbi.nlm.nih.gov/snp/. The datasets used and/or analyzed during the study are available from the corresponding author on reasonable request.

## References

[1] Hillemacher T, Bleich S (2008). Neurobiology and treatment in alcoholism–recent findings regarding Lesch’s typology of alcohol dependence. *Alcohol and Alcoholism*.

[2] Goodman A (2008). Neurobiology of addiction. An integrative review. *Biochemical Pharmacology*.

[3] Gonzales RA, Jaworski JN (1997). Alcohol and glutamate. *Alcohol Health and Research World*.

[4] Dodd PR, Beckmann AM, Davidson MS, Wilce PA (2000). Glutamate-mediated transmission, alcohol, and alcoholism. *Neurochemistry International*.

[5] Terranova C, Tucci M, Forza G, Barzon L, Palù G, Ferrara SD (2010). Alcohol dependence and glutamate decarboxylase gene polymorphisms in an Italian male population. *Alcohol*.

[6] Xia Y, Wu Z, Ma D, Tang C, Liu L, Xin F (2014). Association of single-nucleotide polymorphisms in a metabotropic glutamate receptor GRM3 gene subunit to alcohol-dependent male subjects. *Alcohol and Alcoholism*.

[7] Kranzler HR, Gelernter J, Anton RF, Arias AJ, Herman A, Zhao H (2009). Association of markers in the 3’ region of the GluR5 kainate receptor subunit gene to alcohol dependence. *Alcoholism, Clinical and Experimental Research*.

[8] Malenka RC, Nicoll RA (1999). Long-term potentiation-a decade of progress?. *Science*.

[9] Bliss TV, Collingridge GL (1993). A synaptic model of memory: long-term potentiation in the hippocampus. *Nature*.

[10] Trujillo KA, Akil H (1995). Excitatory amino acids and drugs of abuse: a role for N-methyl-D-aspartate receptors in drug tolerance, sensitization and physical dependence. *Drug and Alcohol Dependence*.

[11] Krystal JH, Petrakis IL, Mason G, Trevisan L, D’Souza DC (2003). N-methyl-D-aspartate glutamate receptors and alcoholism: reward, dependence, treatment, and vulnerability. *Pharmacology & Therapeutics*.

[12] Wang YT, Salter MW (1994). Regulation of NMDA receptors by tyrosine kinases and phosphatases. *Nature*.

[13] Salter MW, Pitcher GM (2012). Dysregulated Src upregulation of NMDA receptor activity: a common link in chronic pain and schizophrenia. *The FEBS Journal*.

[14] Yu XM, Askalan R, Keil GJ, Salter MW (1997). NMDA channel regulation by channel-associated protein tyrosine kinase Src. *Science*.

[15] Salter MW, Kalia LV (2004). Src kinases: a hub for NMDA receptor regulation. *Nature Reviews. Neuroscience*.

[16] Miyakawa T, Yagi T, Kitazawa H, Yasuda M, Kawai N, Tsuboi K (1997). FYN-kinase as a determinant of ethanol sensitivity: relation to NMDA-receptor function. *Science*.

[17] Boehm SL, Peden L, Chang R, Harris RA, Blednov YA (2003). Deletion of the FYN-kinase gene alters behavioral sensitivity to ethanol. *Alcoholism, Clinical and Experimental Research*.

[18] Schumann G, Rujescu D, Kissling C, Soyka M, Dahmen N, Preuss UW (2003). Analysis of genetic variations of protein tyrosine kinase FYN and their association with alcohol dependence in two independent cohorts. *Biological Psychiatry*.

[19] Pastor IJ, Laso FJ, Inés S, Marcos M, González-Sarmiento R (2009). Genetic association between -93A/G polymorphism in the FYN kinase gene and alcohol dependence in Spanish men. *European Psychiatry*.

[20] Ishiguro H, Saito T, Shibuya H, Toru M, Arinami T (2000). Mutation and association analysis of the FYN kinase gene with alcoholism and schizophrenia. *American Journal of Medical Genetics*.

[21] Heath AC, Slutske WS, Madden PA, Wilsnack RW, Wilsnack SC (1997). Gender differences in the genetic contribution to alcoholism risk and to alcohol consumption patterns. *Gender and alcohol: Individual and social perspectives*.

[22] Thomasson HR (1995). Gender differences in alcohol metabolism. Physiological responses to ethanol. *Recent Developments in Alcoholism*.

[23] Schulte MT, Ramo D, Brown SA (2009). Gender differences in factors influencing alcohol use and drinking progression among adolescents. *Clinical Psychology Review*.

[24] Li TK, Yin SJ, Crabb DW, O’Connor S, Ramchandani VA (2001). Genetic and environmental influences on alcohol metabolism in humans. *Alcoholism, Clinical and Experimental Research*.

[25] Nolen-Hoeksema S (2004). Gender differences in risk factors and consequences for alcohol use and problems. *Clinical Psychology Review*.

[26] Wakabayashi I, Araki Y (2010). Influences of gender and age on relationships between alcohol drinking and atherosclerotic risk factors. *Alcoholism, Clinical and Experimental Research*.

[27] Prescott CA, Aggen SH, Kendler KS (1999). Sex differences in the sources of genetic liability to alcohol abuse and dependence in a population-based sample of U.S. twins. *Alcoholism, Clinical and Experimental Research*.

[28] Town T, Abdullah L, Crawford F, Schinka J, Ordorica PI, Francis E (1999). Association of a functional mu-opioid receptor allele (+118A) with alcohol dependency. *American Journal of Medical Genetics*.

[29] Chan AW (1986). Racial differences in alcohol sensitivity. *Alcohol and Alcoholism*.

[30] Dick DM, Bierut LJ (2006). The genetics of alcohol dependence. *Current Psychiatry Reports*.

[31] Luczak SE, Wall TL, Cook TAR, Shea SH, Carr LG (2004). ALDH2 status and conduct disorder mediate the relationship between ethnicity and alcohol dependence in Chinese, Korean, and White American college students. *Journal of Abnormal Psychology*.

[32] Schinka JA, Town T, Abdullah L, Crawford FC, Ordorica PI, Francis E (2002). A functional polymorphism within the mu-opioid receptor gene and risk for abuse of alcohol and other substances. *Molecular Psychiatry*.

[33] Zahratka JA, Shao Y, Shaw M, Todd K, Formica SV, Khrestian M (2017). Regulatory region genetic variation is associated with FYN expression in Alzheimer’s disease. *Neurobiology of Aging*.

[34] Anbarasu A, Kundu A (2012). In silico study of Alzheimer’s disease in relation to FYN gene. *Interdisciplinary Sciences, Computational Life Sciences*.

[35] Liu W, Zhao J, Lu G (2016). miR-106b inhibits tau phosphorylation at Tyr18 by targeting FYN in a model of Alzheimer’s disease. *Biochemical and Biophysical Research Communications*.

[36] Bekris LM, Millard S, Lutz F, Li G, Galasko DR, Farlow MR (2012). Tau phosphorylation pathway genes and cerebrospinal fluid tau levels in Alzheimer’s disease. *American Journal of Medical Genetics. Part B, Neuropsychiatric Genetics*.

[37] Yang K, Belrose J, Trepanier CH, Lei G, Jackson MF, MacDonald JF (2011). FYN, a potential target for Alzheimer’s disease. *Journal of Alzheimer’s Disease*.

[38] Saito M, Chakraborty G, Mao RF, Paik SM, Vadasz C, Saito M (2010). Tau phosphorylation and cleavage in ethanol-induced neurodegeneration in the developing mouse brain. *Neurochemical Research*.

[39] Hardie TL, Moss HB, Lynch KG (2008). Sex differences in the heritability of alcohol problems. *The American Journal on Addictions*.

[40] Devaud LL, Morrow AL (1999). Gender-selective effects of ethanol dependence on NMDA receptor subunit expression in cerebral cortex, hippocampus and hypothalamus. *European Journal of Pharmacology*.

